# 3D-AI mouse behavior analysis system has the capability to detect abnormalities in R6/1 model mice with Huntington’s disease during the pre-symptomatic phase

**DOI:** 10.3389/fpsyt.2026.1749543

**Published:** 2026-03-10

**Authors:** Shida Zhou, Xiaoyu Wang, Yu Tian Wang

**Affiliations:** 1 Translational Research Center for the Nervous System, the Brain Cognition and Brain Disease Institute, Shenzhen Institutes of Advanced Technology, Chinese Academy of Sciences, Shenzhen, China; 2 University of Chinese Academy of Sciences, Beijing, China; 3 Shenzhen Key Laboratory of Neuropsychiatric Modulation, Shenzhen-Hong Kong Institute of Brain Science, Shenzhen Institutes of Advanced Technology, Chinese Academy of Sciences, Shenzhen, China; 4 Guangdong Provincial Key Laboratory of Brain Connectome and Behavior, the Brain Cognition and Brain Disease Institute, Shenzhen Institutes of Advanced Technology, Chinese Academy of Sciences, Shenzhen, China; 5 Department of Neuroscience, Faculty of Life and Health Sciences, Shenzhen University of Advanced Technology, Shenzhen, China; 6 Fudan-SANS Neuroscience Center, Fudan University, Shanghai, China

**Keywords:** 3D motion capture, animal disease model, dyskinesia, Huntington’s disease, premanifest phase, psychiatric disorder

## Abstract

**Introduction:**

Huntington's disease (HD), a dominantly inherited neurodegenerative disorder caused by CAG repeat expansions in the HTT gene, manifests with progressive motor dysfunction, cognitive decline, and psychiatric disturbances. While current transgenic mouse models recapitulate key pathological features, they exhibit rapid disease progression, and early behavioural phenotypes are not analyzed comprehensively to understand their progression.

**Methods:**

We employed a high-resolution 3-dimensional motion capture and unsupervised machine learning to dissect behavioral dynamics in the R6/1 HD mouse model at 8 weeks of age, a stage analogous to human pre-diagnostic HD.

**Results:**

Through unsupervised learning-based clustering analysis, we identified 40 major movement categories in mice. Using a subsequent supervised learning approach, we recognized 13 fundamental spontaneous behavioral movements and identified disrupted behavioral modules in R6/1 mice, including reduced locomotor fraction, increased pausing frequency, and altered exploratory patterns. Our key findings revealed that HD mice exhibited reduced velocity and increased stride length during running and trotting behaviors, mirroring bradykinesia and gait abnormalities observed in HD patients. These mice also showed preferential exploration of the peripheral zone and decreased sniffing frequency, which might suggest that they have displayed behaviors analogous to anxiety or depression.Furthermore, an escalating frequency of pausing was observed over 30-minute sessions, suggesting early-onset motor fatigue. Additionally, lower behavioral entropy and fewer transitions from exploratory or maintenance states to locomotion were detected, pointing to executive dysfunction. A LDA classifier integrating these core behavioral metrics achieved an AUC of 0.917, surpassing the performance of traditional coarse motor assessments.

**Conclusion:**

These results establish precision behavioral analytics as a sensitive platform for detecting premanifest HD pathology, providing a framework for evaluating presymptomatic therapeutics and scientific base for developing early diagnostic and treatment strategies for HD.

## 1 Introduction

Huntington’s disease (HD), a dominantly inherited neurodegenerative disorder caused by CAG repeat expansions in the HTT gene, remains a paradigmatic challenge in translational neuroscience (1). Characterized by rapid progressive motor dysfunction, cognitive decline, and psychiatric disturbances, HD critically needs early diagnostic biomarkers and disease-modifying interventions (2, 3). While the identification of HTT mutation carriers via genetic testing has improved prognostic accuracy, the absence of effective early diagnostic strategies and therapies underscores the urgency to dissect premanifest pathophysiology using physiologically relevant models.

Transgenic mouse models, such as R6/2, recapitulate key HD features but suffer from rapid disease progression and non-specific phenotypes, limiting their utility for studying early-stage phenotypes and mechanisms (4, 5). Full-length knock-in models better mimic human pathology, but lack sensitivity for detecting subtle behavioral abnormalities in preclinical stages (6, 7). Recent advances in conventional behavioral analytics, however, have highlighted the limitations of coarse motor assays in resolving early-stage dysfunctions. J. Ouwerkerk et al. reported a novel multiple machine learning models were able to outperform the conventional Langbehn formula, and driving capability was predicted with an accuracy of 85.2% (8). This finding indicated that via the development of novel methods, it become feasible to detect abnormal conditions in patients during the earlier stages of HD.

Recent technological breakthroughs in unsupervised machine learning and high-resolution motion capture systems have revolutionized behavioral neuroscience. Hierarchical frameworks, such as the Behavior Atlas Lite platform, enable decomposition of complex behaviors into quantifiable modules (e.g., locomotion, grooming, exploration), revealing subtle phenotypes undetectable by manual scoring (9). Inspired by the natural structure of animal behavior, K. Huang et al. (10). developed a parallel multi-layer framework to capture hierarchical behavioral dynamics and introduced an objective metric to map these behaviors into a structured feature space. By leveraging an efficient multi-view 3D animal motion capture system, K. Huang et al. characterized three-dimensional kinematic features, monitored spontaneous behaviors, and achieved automated identification of behavioral phenotypes in animal disease models. Three-dimensional kinematic gait analysis holds promise as a digital biomarker for identifying neuropathologies, tracking disease progression, and providing high-resolution outcome measures to evaluate the efficacy of neurorehabilitation through detailed characterization of the underlying mechanisms of gait impairments (10, 11). Such approaches align with the Human Phenotype Ontology initiative, prioritizing granular behavioral endophenotypes for clinical translation.

Thus, this Three-Dimensional Kinematic Gait Analysis developed by Huang et al. would be more sensitive in detecting subtle behavioral changes and thereby able to uncover early pathophysiological signatures at a much early stage of disease progression in HD mice (7, 12). To test our reasoning, here, we leverage the R6/1 transgenic mice model, which expresses a humanized HTT gene with 115 CAG repeats and exhibits delayed symptom onset at the age of later than ~12 weeks. We employed this system at the age of 8 weeks, the proposedly equivalent of human HD premanifest stages. We hypothesize that precision behavioral analytics of clustering fine-grained movements combined with machine-learning classifiers will reveal disrupted behavioral dynamics, which can serve as sensitive biomarkers for developing early diagnostic and therapeutic strategies.

By integrating high-resolution behavioral phenotyping with computational biology, we define the spatiotemporal evolution of subtle motor and exploratory deficits in R6/1 mice; identify multimodal behavioral signatures predictive of disease progression; and thereby establish a framework for evaluating premanifest therapeutic efficacy in alignment with emerging ISS-HD staging criteria. Our research addresses two critical gaps in HD research: first, the disconnect between preclinical models and human presymptomatic pathophysiology; and second, the lack of sensitive biomarkers for early-stage diagnosis. Ultimately, this work will bridge the translational gap in HD research and provide a novel paradigm for the early diagnosis of HD.

## 2 Materials and methods

### 2.1 Experimental animals

The experimental animals used in this study were 8-week-old male R6/1 transgenic mice (Jackson Laboratory stock number: B6.Cg-Tg(HDexon1)61Gpb/J, strain number 006471), obtained from Wuhan Youdu Biotechnology Co., Ltd. (Experimental Animal Production License No.: SCXK (E) 2021-0025). A total of 19 transgenic mice were used, along with 19 wild-type (WT) littermates that served as controls. All procedures were approved by the Animal Care and Use Committee of Shenzhen Institutes of Advanced Technology, Chinese Academy of Sciences (SIAT-IACUC-230607-NS-WYT-A2284-03). Upon arrival at the laboratory, the animals were allowed to acclimate to the housing environment for one week before experiments start.

The schematic diagram of the research device and the timeline of 3D behavior data acquisition and processing are presented in **Supplementary Figures 1A, B**.

1. Body Weight Monitoring:

The body weight of the mice was recorded at eight weeks of age.

2. Rotarod Test: The experiment protocol includes a training phase of 2 trials followed by a testing phase of 5 trials. In the training phase, the rotarod speed gradually increased from 0 to 20 revolutions per minute (rpm) over 60 seconds and was maintained for 180 seconds. In the testing phase, the rotarod speed increased from 0 to 45 rpm over 180 seconds and was maintained at 45 rpm for 120 seconds. The intertrial interval was 3 minutes. The latency to fall was recorded for each animal.

3. Balance Beam Test: The apparatus consisted of a wooden beam positioned 50 cm above ground. The beam had two segments with different cross-sectional widths: 2.5 cm and 1.0 cm. A starting platform was located at one end and a goal box at the other end, separated by 50 cm. The time for each mouse to traverse the beam was recorded.

4. The pole-climbing experiment: The testing apparatus consisted of a wooden stick, 60 cm in length and 2 cm in diameter, vertically fixed to the ground. The surface of the stick was wrapped with gauze to ensure adequate grip. The time required for each mouse to descend from the top to the bottom was recorded.

5. Elevated plus maze experiment: At 8 weeks of age, anxiety-like behaviors were assessed using the elevated plus maze. The maze was elevated 1 meter above ground and consisted of two open arms (30 cm×5 cm), two closed arms (30 cm×5 cm), and a central area (5 cm×5 cm) connecting all four arms. Each mouse was placed in the central area facing an open arm. The maze was cleaned with 75% alcohol between trials. The number of open arm entries and time spent in open arms were recorded using Anymaze software. The percentage of open arm entries was calculated as: (open arm entries/total entries) × 100%.

6. 3D behavior data acquisition and processing

This experiment was performed as based on our previous studies (10, 13, 14).

Three-dimensional behavioral data were collected using a 3D-AI mouse behavior analysis system (BA-3D Mouse 01; Guangdong Bayone Biotech CO., Ltd.). The system includes an integrated hardware enclosure that houses an open-field arena, cameras, and the main computing unit. A cylindrical open-field arena was positioned inside the enclosure. During experiments, mice were individually placed in the open field, and their freely moving behaviors were synchronously recorded by four cameras.

The dimensions of the hardware enclosure were 130×130×90 cm. Four Intel RealSense D435 cameras were mounted on the four vertical pillars of the enclosure and carefully adjusted to ensure complete coverage of the open-field arena and the full movement range of the mouse. The open field had a cylindrical structure with a diameter of 50 cm and a height of 40 cm. The arena walls were constructed from transparent material to enable unobstructed multi-view synchronous recording.

### 2.2 Camera calibration

After adjusting the camera viewpoints, multi-camera position calibration was performed using the BehaviorAtlas Capture Mouse software (V1.0.1; Guangdong Bayone Biotech CO., Ltd.). During calibration, 185 pairs of calibration images containing an 11× 8 checkerboard pattern were acquired within the overlapping fields of view of the primary camera and each of the three secondary cameras, and the corresponding calibration parameters were computed.

### 2.3 Data collection

After determining the camera position parameters and completing pre-experimental preparations (including lighting adjustment and resolution and frame rate settings), mouse behavioral data were synchronously recorded from four viewpoints using the above acquisition software. The video frame rate was set to 30 fps with a spatial resolution of 960×540 pixels, and each mouse was continuously recorded for 30 minutes.

### 2.4 3D fine-grained behavioral data processing

#### 2.4.1 Two-dimensional pose tracking

The collected data were processed using the BehaviorAtlas Analyzer Mouse software (V2.0.0; Guangdong Bayone Biotech CO., Ltd.). The software incorporates a built-in two-dimensional pose estimation model that automatically tracks mouse body key points across all camera views. A total of 16 body key points were tracked, including the nose, left ear, right ear, neck, left front limb, right front limb, left hind limb, right hind limb, left front claw, right front claw, left hind claw, right hind claw, back, root tail, middle tail and tip tail. This process generated two-dimensional coordinate data for each key point.

#### 2.4.2 Three-dimensional skeleton reconstruction

Three-dimensional skeleton reconstruction was performed in the BehaviorAtlas Analyzer Mouse software based on the multi-camera calibration parameters obtained prior to data acquisition, in combination with the key-point coordinates derived from two-dimensional pose tracking. For each sample, a data matrix of size N×48 (16 key points×3 spatial coordinates) was generated, where N represents the number of video frames. Each row corresponds to a single video frame, and every three consecutive columns represent the x, y, and z coordinates of an individual body key point. The reconstructed three-dimensional behavioral data were saved in.h5 format.

### 2.5 Unsupervised behavior classification

Following three-dimensional reconstruction, mouse behaviors were automatically classified using the same software based on an unsupervised clustering algorithm, which by default categorized behaviors into 40 distinct behavioral phenotypes. After classification, the software automatically segmented video clips corresponding to each behavior category from the recordings of camera view 1 and saved them into separate folders according to behavior type for subsequent manual annotation and correction.

### 2.6 Behavior annotation, merging, and correction

From the video clip folders corresponding to the 40 behavioral phenotypes, 100 video clips were randomly selected from each phenotype and subjected to continuous observation. Manual movement annotation and naming were performed based on previous studies on three-dimensional modeling of spontaneous mouse behavior and hierarchical behavioral analysis (9, 10, 15), with additional reference to the definitions and descriptions of typical mouse behaviors provided by the Stanford Mouse Ethogram. Given that some behavioral phenotypes exhibited highly similar movement patterns, biologically related behaviors were merged according to their functional significance. Ultimately, 13 basic spontaneous movement types were identified: Running, Trotting, Walking, Stepping, Right turning, Left turning, Sniffing, Rising, Rearing, Climbing up, Jumping, Grooming, and Pausing.

To further improve the accuracy of behavior recognition, several behavior labels were refined according to predefined rules (9, 15), as detailed below (**Table 1**):

**Table 1:** The predefined rules used for revising behavioral labels.

Behavior label	Revision
Jumping	Back height (back z) continuously>100 mm for at least 2 frames
Pausing	Movement speeds of both nose tip and back<15 mm/s during behaviors such as Sniffing, Stepping, Grooming, Turning, Rising, and Trotting
Climbing up	Front paws near the edge of the cage wall (>0.8×radius) and back height>40 mm; otherwise classified as Rising
Rising	Original Rising movements not meeting Climbing up criteria
Rearing	Among movements originally labeled as Rising, neck height (neck z)>50 mm and lasts≥15 frames
Grooming	Movements originally labeled as Rearing, Climbing, Rising, or Turning and lasting>300 frames (10 s)

Jumping: A behavior was classified as jumping when the back height (back z) continuously exceeded 100 mm for at least two consecutive frames.

Pausing: During behaviors such as sniffing, stepping, grooming, turning, rising, and trotting, a segment was classified as pausing when the movement speeds of both the nose tip and the back were below 15 mm/s.

Differentiation between Climbing up and Rising: If the front paws were close to the edge of the arena (distance > 0.8×arena radius) and the back height exceeded 40 mm, the behavior was classified as climbing; otherwise, it was categorized as rising.

Rearing: Among behaviors initially labeled as rising, those with a neck height (neck z) exceeding 50 mm for at least 15 consecutive frames were relabeled as rearing.

Grooming: Among behaviors originally labeled as rearing, climbing, rising, or turning, those with a duration exceeding 300 frames (i.e., 10 s) were relabeled as grooming.

Finally, based on similarities in movement features, the 13 behaviors were further grouped into four behavioral categories for subsequent analyses.

### 2.7 3D fine-grained behavioral data analysis

#### 2.7.1 Inter-group classification analysis based on movement features

After completing movement annotation and merging similar behavioral phenotypes, the occurrence frequencies of the 13 behavior types were quantified for wild-type (WT) and Huntington’s disease (HD) mice. Linear discriminant analysis (LDA) was applied to classify the two groups, and leave-one-out cross-validation (LOOCV) was used to evaluate model generalization performance. Evaluation metrics included the confusion matrix, macro-averaged F1 score (macro-F1), and area under the curve (AUC).

Furthermore, four behavioral phenotypes exhibiting significant inter-group differences (Running, Walking, Trotting, and Pausing) were selected. Their proportional contributions were calculated, and LDA classification was repeated based on these key phenotypes, using the same LOOCV evaluation strategy and performance metrics as described above.

#### 2.7.2 Extraction of kinematic parameters

Movement speed: Based on the trajectories of the back key point projected onto the XY plane, frame-by-frame instantaneous velocity was calculated using the displacement between adjacent frames. A five-frame median filter (approximately 167 ms at 30 fps) was subsequently applied to the velocity data to reduce high-frequency noise introduced by pose estimation errors, tracking jittering, or abnormal frames. Using the processed velocity data, velocity distributions of WT and HD mice during running, trotting, walking, and stepping behaviors were visualized, and statistical differences among behavioral states were compared.

Gait parameters: For each motion segment, the distance between the left forelimb and hindlimb was first calculated using frame-by-frame three-dimensional coordinate data. A moving average smoothing was then applied to this distance sequence to reduce high-frequency noise caused by pose estimation errors or tracking jitter. Local peaks and valleys were subsequently detected in the smoothed sequence, corresponding to the maximum extension and maximum contraction of the limb during the gait cycle, respectively. The stride length of a single step was defined as the average difference between each peak and its adjacent valley, reflecting the spatial extension amplitude of the limb during one step.

Behavioral angle: Angles were calculated based on the frame-by-frame three-dimensional coordinates of the nose, neck, and back key points to represent the upper body posture, and based on the nose, back, and root tail key points to represent the lower body posture. Analysis of these angles was used to quantify postural changes during sniffing, rising, rearing, and climbing behaviors, thereby enabling a more in-depth investigation of behavioral dynamics.

Body stretch ratio: To quantify changes in body posture between genotypes (WT and HD), the body stretch ratio was defined. Based on three-dimensional pose estimation data, Euclidean distances between the back key point and seven peripheral body key points (nose, neck, left front limb, right front limb, left hind limb, right hind limb, root tail) were calculated, and the sum of these seven distances was defined as the total body distance for each frame.

To achieve cross-individual normalization, the average total body distance of WT mice during sniffing behavior was used as the reference value (100%). The body stretch ratio for each frame was calculated by dividing the current total body distance by this reference value and multiplying by 100%. This metric was used to assess the degree of body stretching or curling in WT and HD mice during behaviors such as grooming and pausing.

#### 2.7.3 Analysis of dynamic changes in behavior ratios within time windows

Each sample was recorded for 30 minutes, and the experimental period was divided into three time windows: 0–10 min, 10–20 min, and 20–30 min. The proportions of the 13 behaviors and the four movement sets were calculated for each time window. Bar charts and pie charts were used to visually compare behavioral proportions between the WT and HD groups across different time periods.

Behavioral spatial distribution analysis based on radial partitioning

The radius of the open-field arena was 25 cm. Using the center of the arena floor as the origin, the arena was divided into a central zone (0-7.5 cm), a middle zone (7.5–15 cm), and a peripheral zone (15–25 cm) based on radial distance. Residence time and behavioral proportions within each zone were calculated. Rose plots and statistical tests were used to compare differences in spatial behavioral distributions between the WT and HD groups.

### 2.8 Behavioral similarity analysis

The 30-minute recording of each sample was divided into five consecutive time windows (0-6, 6-12, 12-18, 18-24, and 24–30 min). Within each time window, occurrences of the 13 behavioral subtypes were counted for each sample, yielding a behavior count matrix of size 18×13 for each group per time window. A total of five time-window matrices were obtained for both the WT and HD groups.

Based on these behavior count matrices, population-level behavioral similarity was assessed between any two time windows within each group. Specifically, samples in the two corresponding 18×13 matrices were pairwise matched, and Pearson correlation coefficients were calculated to generate an 18×18 similarity matrix. The average correlation coefficient of this matrix was used as the population behavioral similarity index for the corresponding time-window pair. Ultimately, 5×5 time-window behavioral similarity matrices were obtained for the WT and HD groups and visualized as heatmaps. The diagonal elements represent population behavioral similarity within the same time window, whereas off-diagonal elements represent similarity between different time windows; diagonal values are not necessarily equal to 1.

### 2.9 Movement state transition analysis

#### 2.9.1 Calculation of movement state transition probability

State transition probability was used to describe the likelihood of transitioning from one movement state to another within a behavioral time series. Under the first-order Markov assumption, the proportion of transition events that start from movement i and end at movement j was calculated. The transition probability was defined as:


$$P(j|i)=\frac{N(i\to j)}{\sum_{k}N(i\to k)}$$

Among them, N(i→j) represents the actual number of occurrences of the transition from movement i to movement j, while the denominator represents the total number of times that movement i transitions to all other movements. As a result, the transition probability matrix is obtained.

#### 2.9.2 Movement transition analysis at three hierarchical levels

A. State transitions among the 13 basic movement categories (fine-grained transition network)

In this analysis, network nodes represent the 13 basic movement types, and edge thickness indicates the magnitude of the corresponding transition probabilities. This level preserves the most fine-grained behavioral classification and was used to systematically compare differences in specific movement-switching patterns between experimental groups. The results were visualized as transition networks for each group, and a heatmap depicting differences in movement transition probabilities between the HD and WT groups was generated.

B. Connection properties of the top four dominant nodes

Furthermore, the four core movement nodes with the highest connectivity were selected to analyze their transition relationships with other movements. Node connectivity was quantified by counting the number of direct connections between each node and other nodes in the network. This analysis was used to identify key movements that function as hubs within the overall behavioral sequence and to characterize their organizational roles during transitions between behavioral states. Results were visualized using network diagrams.

C. Transitions among four major behavioral clusters (higher-order transitions)

At a higher level of analysis, the 13 basic movements were further grouped into four functional behavioral clusters: Locomotion, Exploration, Maintenance, and Nap. State transition probabilities were recalculated based on this categorization to assess large-scale reorganization of behavioral strategies and alterations in high-level behavioral organization in HD model mice. The results were visualized using transition networks for each group, along with heatmaps illustrating differences in transition probabilities between the HD and WT groups.

### 2.10 Statistical analysis

In this study, the statistical significance level was set at P< 0.05. All measurement data were expressed as mean ± standard deviation (x ± s). Prior to analysis, the data were subjected to normality tests (Shapiro-Wilk test) and tests for homogeneity of variance. If the data met the assumptions of normality and homogeneity of variances, an independent samples t-test was used for comparisons between two groups. If the assumption of normality was not satisfied, the Mann-Whitney U test was employed. For categorical variables, the chi-square test was applied. Specifically, for the movement fraction and movement frequency indicators, the Shapiro-Wilk test was conducted first. If these data did not follow a normal distribution, the Mann-Whitney U test was used to assess inter-group differences. To control for the increased risk of false positives due to multiple comparisons, the Bonferroni-Dunn correction was applied to the obtained *P* values, and the corrected significance level was used to determine statistical significance. *<0.05, **<0.01, ***<0.001.

## 3 Results

### 3.1 Traditional behavioral assessments in HD mice models

**Figure 1:**
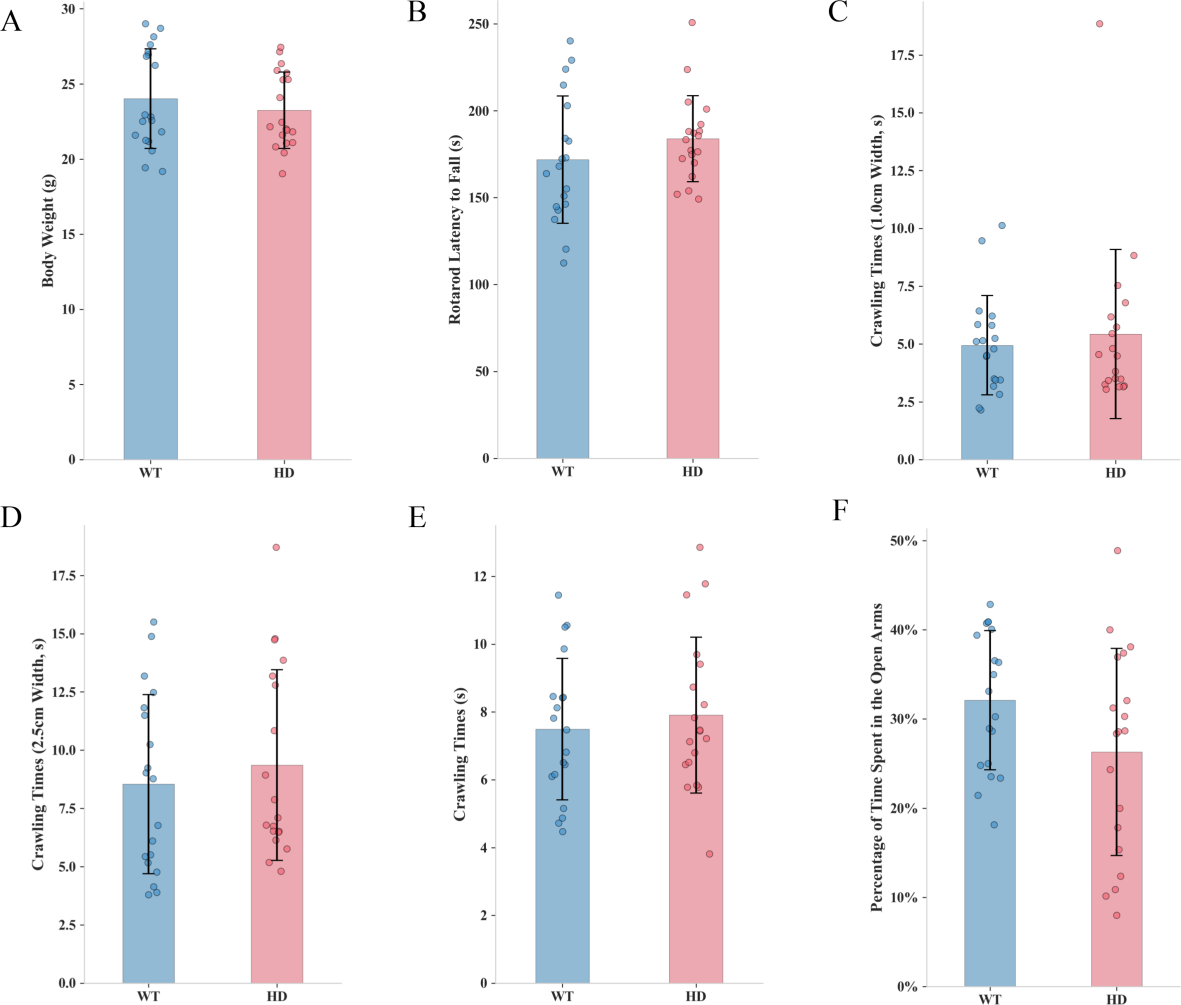
Traditional behavioral assessments in HD mice models. **(A)** Body weight (g). **(B)** Rotarod latency to fall (s). **(C)** Crawling time on the balance beam (2.5 cm width, s). **(D)** Crawling time on the balance beam (1 cm width, s). **(E)** Crawling time on the vertical climbing pole (s). **(F)** Percentage of time spent in the open arms during the elevated plus maze test. (n=19, *<0.05, **<0.01, ***<0.001.) All data were initially subjected to the normality test (Shapiro-Wilk test) and the homogeneity of variance test. In the event that the data satisfied the conditions of normality and homogeneity of variance, the independent-samples t-test was employed for comparison between the two groups. Conversely, if the normality assumption was not fulfilled, the Mann-Whitney U test was utilized.

Body weight measurement, latency to fall in the rotarod test, performance on the balance beam test, and related behavioral assays are commonly used traditional methods for assessing HD in mice models. In this study, we conducted these standard behavioral tests in 8-week-old WT and R6/1 transgenic mice. Our results revealed that, at 8 weeks of age-corresponding to the early prodromal stage of human HD-R6/1 mice exhibited no significant differences compared to WT controls in body weight (**Figure 1A**), rotarod performance (**Figure 1B**), variable widths of balance beam traversal (**Figures 1C, D**), vertical pole climbing ability (**Figure 1E**), or percentage of time spent in the open arms of the elevated plus maze (**Figure 1F**). These results are in a good agreement with previous studies that with conventional behavioral assessments these mice showed no detectable abnormalities at this age (16, 17).

### 3.2 Spontaneous behavioral characteristics in HD mice models

**Figure 2:**
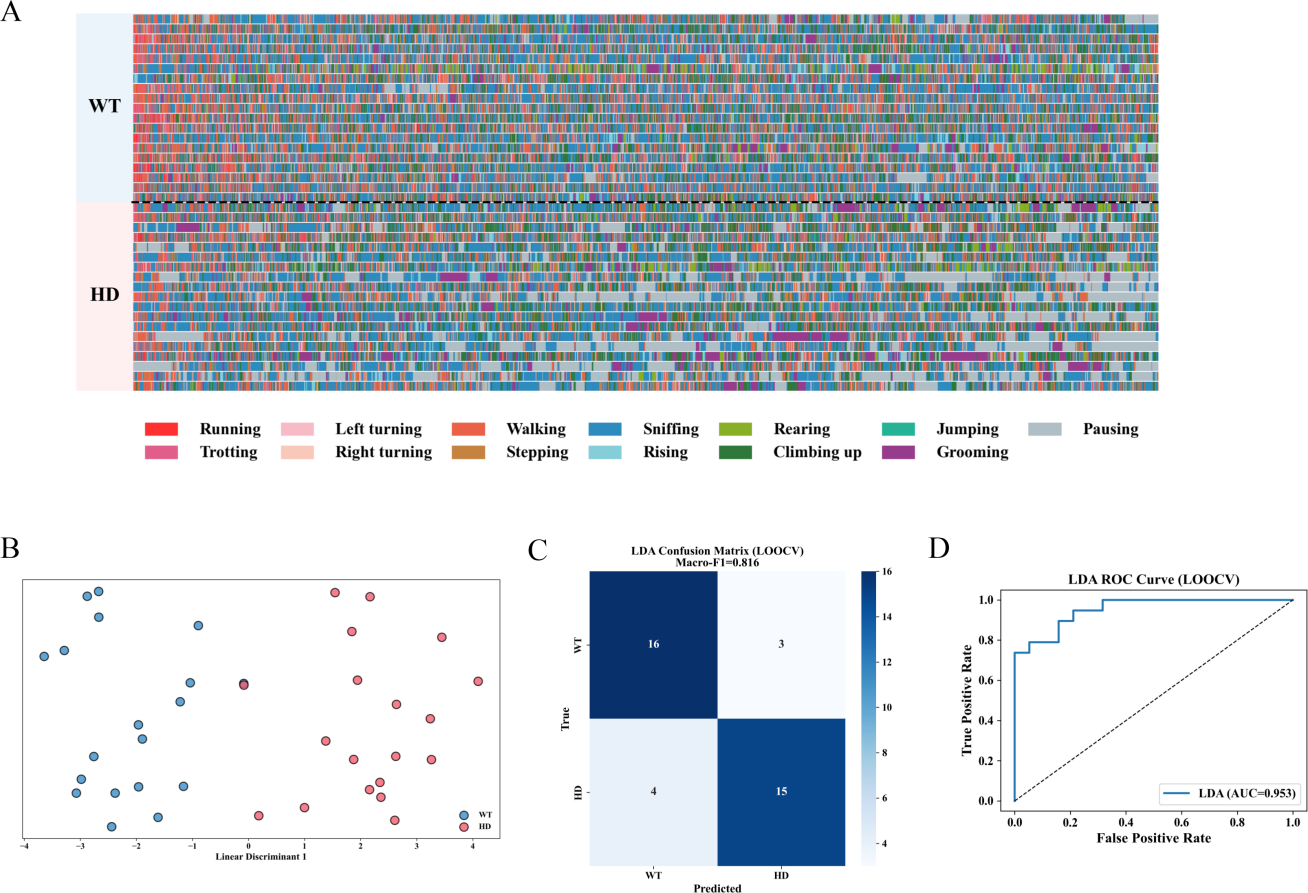
Spontaneous behavioral characteristics of HD mice. **(A)** Behavioral atlas of mice. **(B)** LDA classification (categorized by the number of simple movements). **(C)** LDA confusion matrix. **(D)** LDA ROC curve. (n=19). In this study, Python 3.8 was utilized for data analysis, and the scikit-learn library was applied to implement Linear Discriminant Analysis (LDA). The input data consisted of the counts of behavior segments statistically derived from the 3D behavior annotation results. A total of 13 types of behaviors were incorporated as analysis features. All features underwent Z-score normalization prior to analysis. LDA solved by Singular Value Decomposition (SVD) was adopted as a supervised classification method to differentiate between WT and HD mice, and one-dimensional LDA projection was employed for visualization. The performance of the model was assessed through Leave-One-Out Cross-Validation (LOOCV), and the evaluation metrics encompassed Macro-F1, confusion matrix, and ROC-AUC.

The fine-grained behavioral analysis of mice relies on a multi-view 3D motion capture system that reconstructs dynamic postures by tracking 16 skeletal key points. As such, we reasoned this new approach may be more sensitive than conventional behavioral assessments used above and in previous studies, thereby being able to detect some subtle abnormalities in 8-week-old WT and R6/1 transgenic mice. To test this reasoning, we employed this fine-grained behavioral analysis approach to systematically assess the spontaneous behavioral profiles in WT mice and HD mice. The core workflow consists of the following stages: data collection, behavior decomposition, pattern analysis, and application validation (9, 15). The system utilizes unsupervised clustering to identify spontaneous behavioral patterns in mice, followed by manual curation and threshold-based filtering to refine behaviors (**Supplementary Figure 2**). Using this method, mice spontaneous behaviors were classified into 13 distinct categories and subsequently grouped into 4 overarching behavioral clusters (**Table 2**). The analysis results of the temporal distribution of 13 locomotor behaviors in mice over a 30-minute period demonstrated a distinct difference in temporal patterns between the HD and WT groups (**Figure 2A**). Next, we employed the LDA classification method from machine learning to evaluate the model’s ability to differentiate spontaneous behavioral movements between WT and HD mice (9). The results demonstrated that the model could effectively distinguish the spontaneous behavioral patterns of the two mice groups (**Figures 2B, C**). Furthermore, ROC curve analysis confirmed the model’s high classification reliability (**Figure 2D**). The analysis results demonstrate that the fine-grained behavioral analysis method is a reliable and effective approach, capable of early detection of spontaneous behavioral abnormalities in HD mice models that failed to be detected by conventional behavioral analysis.

**Table 2:** Cluster, Movement definition, and action classification.

Clusters	Movements	Defintion
Locomotion	Stepping	The mouse moves forward with short, deliberate steps.
	Walking	The mouse exhibit a relatively low locomotor speed.
	Trotting	The mouse exhibits a relatively high locomotor speed characterized by an intermittent gait pattern.
	Running	The mouse exhibits rapid movement.
	Right turning	The head and body of the mouse exhibits a rightward bend or rotation.
	Left turning	The head and body of the mouse exhibit a leftward bend or rotation.
Exploration	Rising	The mouse raised its body off the ground and stood upright on its hind legs
	Climbing up	The mouse grasps the wall of the box with its forepaws and stands on its hind legs to climb upward.
	Rearing	The mouse stands stably on its hind limbs, with its forepaws not contacting any surface
	Sniffing	mouse explore their environment by actively investigating nearby surfaces and air currents using their noses.
	Jumping	The entire body of the mouse lifts off the ground and moves upward.
Maintance	Grooming	mouse groom themselves by licking their fur, using their forepaws to clean their bodies, or scratching with any limb.
Nap	Pausing	The mouse exhibited a curled posture and remained almost motionless.

### 3.3 Kinetic parameter analysis of the motor behavior of HD mice models

**Figure 3:**
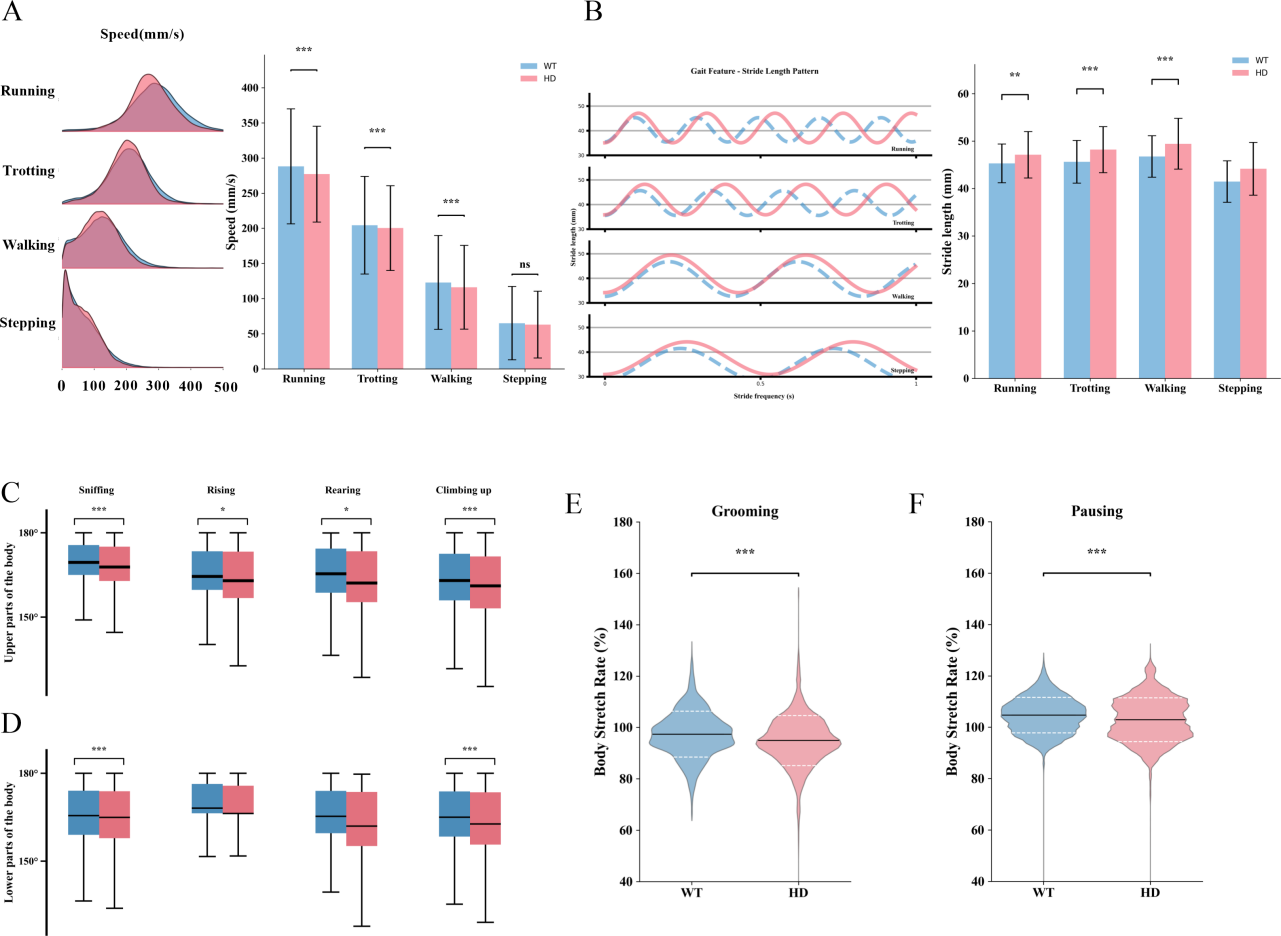
Kinetic parameters of movement in HD mice. **(A)** Distribution of locomotion speeds (Running, Trotting, Walking, Stepping) and statistical analysis. **(B)** Gait dynamics during locomotion and statistical analysis. **(C)** Upper body angles during exploratory behaviors. **(D)** Lower body angles during exploratory behaviors. **(E)** Body stretching angles during grooming. **(F)** Body stretching angles during pause periods. (n=19, *<0.05, **<0.01, ***<0.001.) All data were initially subjected to the normality test (Shapiro-Wilk test) and the homogeneity of variance test. In the event that the data satisfied the conditions of normality and homogeneity of variance, the independent-samples t-test was employed for comparison between the two groups. Conversely, if the normality assumption was not fulfilled, the Mann-Whitney U test was utilized.

To more precisely differentiate locomotor differences in spontaneous behaviors between WT mice and HD mice, we performed a refined kinematic parameter analysis. The results demonstrated that HD mice exhibited reduced speed during Running, Trotting, and Walking (**Figure 3A**). Further gait cycle analysis revealed that, compared to WT mice, HD mice displayed increased stride length and decreased stride frequency across these locomotor patterns (**Figure 3B**). In assessing body angle changes associated with exploratory behaviors, we found that HD mice showed reduced upper body flexion during Sniffing, Rising, Rearing, and Climbing up (**Figure 3C**); additionally, lower body flexion was decreased during Sniffing and Climbing up (**Figure 3D**). Moreover, HD mice exhibited more pronounced body contractions during Grooming and Pausing (**Figures 3E, F**). These findings indicate that the overall locomotor function of early-stage HD mice is already compromised.

The alterations of motor behavior and movement patterns of HD mice models

**Figure 4:**
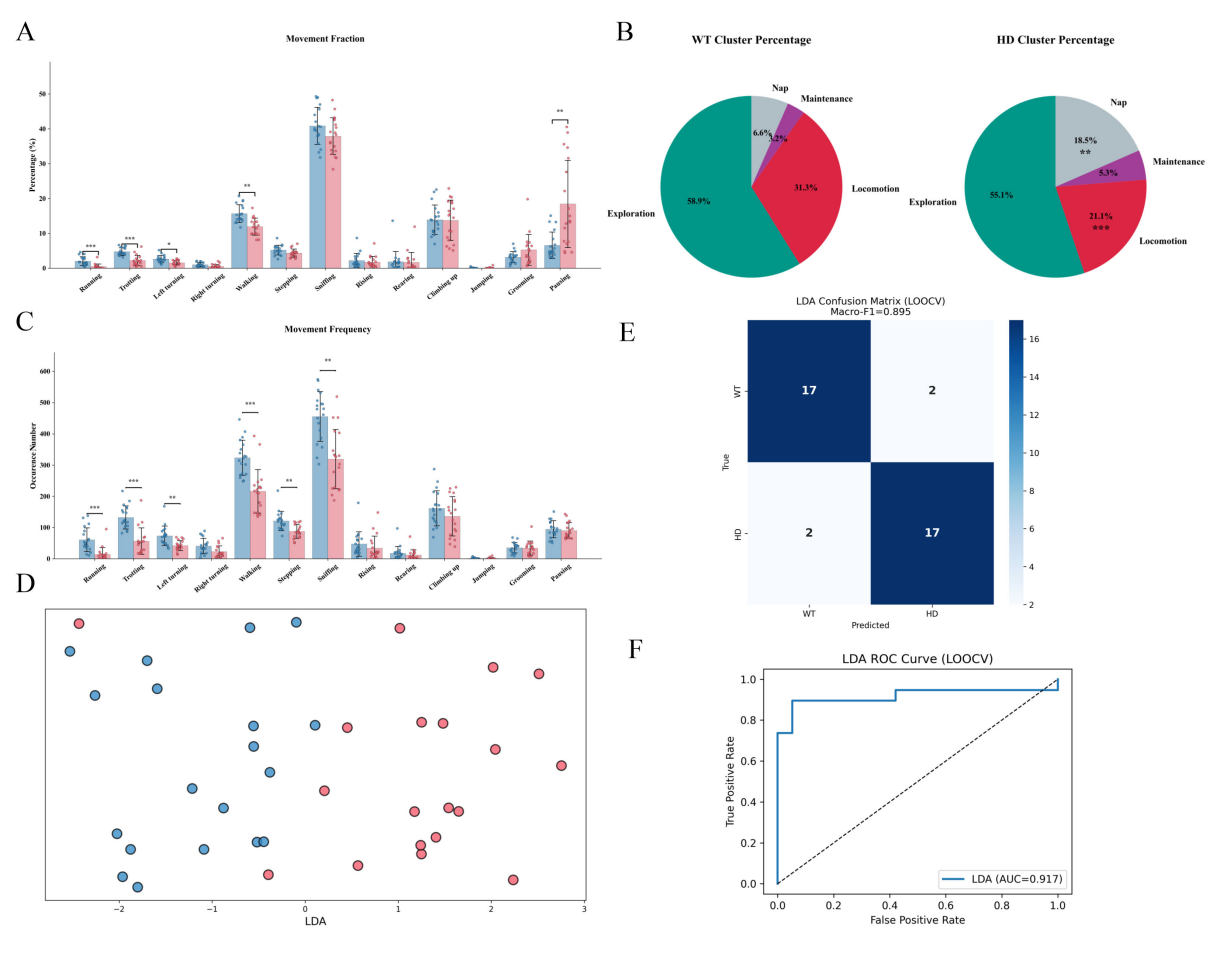
The alterations of motor behavior and movement patterns in HD mice. **(A)** Comparison of movement fraction between HD and WT mice (fraction = number of single movements/total movements). **(B)** Cluster distribution of movement patterns in HD and WT mice. **(C)** Frequency of movements in WT versus HD mice. **(D)** Linear discriminant analysis (LDA) of movement profiles using 5-fold cross-validation. **(E)** LDA confusion matrix. **(F)** LDA ROC curve. (n=19, *<0.05, **<0.01, ***<0.001.) All data were initially subjected to a normality test (Shapiro-Wilk test) and a homogeneity of variance test. If the data satisfied the conditions of normality and homogeneity of variance, one-way analysis of variance and the Bonferroni multiple comparison test were employed for multi-group comparison. For data that were not normally distributed, the non-parametric Mann-Whitney test and Bonferroni-Dunn multiple comparison correction were utilized for statistical analysis. In this study, Python 3.8 was utilized for data analysis, and the scikit-learn library was utilized to implement Linear Discriminant Analysis (LDA). The input feature was the movement fraction, which is defined as the percentage of the number of frames corresponding to each behavior category of each mouse throughout the entire experimental process in relation to the total number of behavior frames. Four types of behaviors, specifically Running, Trotting, Walking, and Pausing, were chosen as the analysis features. All features underwent Z-score normalization prior to analysis. The performance of the model was evaluated via Leave-One-Out Cross-Validation (LOOCV), and Macro-F1, the confusion matrix, and ROC-AUC were employed as the evaluation indicators.

After observing significant alterations in the kinematic parameters of early-stage HD mice, we conducted a systematic analysis of aberrant movement patterns and behavioral repertoires. Mouse behavioral data were first assessed for normality using the Shapiro-Wilk test, followed by inter-group comparisons via the non-parametric Mann-Whitney U test. To mitigate the risk of false positives due to multiple comparisons, p-values were corrected using the Bonferroni method, and statistical significance was determined based on the adjusted threshold. Results revealed that, compared to the WT group, HD mice exhibited significantly lower proportions of Running, Trotting, Left turning, and Walking, while the proportion of Pausing was markedly increased (**Figure 4A**). At the cluster level, HD mice showed reduced Locomotion and elevated Nap proportions (**Figure 4B**). Frequency analysis further demonstrated significant reductions in the occurrence of Running, Trotting, Left turning, Walking, Stepping, and Sniffing in HD mice (**Figure 4C**). Notably, using a LDA model with four movement proportions-Running, Walking, Trotting, and Pausing-and leave-one-out cross-validation approach, WT and HD mice could be effectively classified (**Figure 4D**). The model achieving a Macro-F1 score of 0.895 (**Figure 4E**), and an ROC curve area of 0.917 (**Figure 4F**). These findings indicate that early-stage HD mice display pronounced abnormalities in both movement proportion and frequency. These behavioral metrics demonstrate high classification performance and clearly differentiate early-stage HD mice from normal, highlighting their potential utility as bio-indicators for early detection of HD.

### 3.4 The dynamic characteristics of movement time in HD mice models

**Figure 5:**
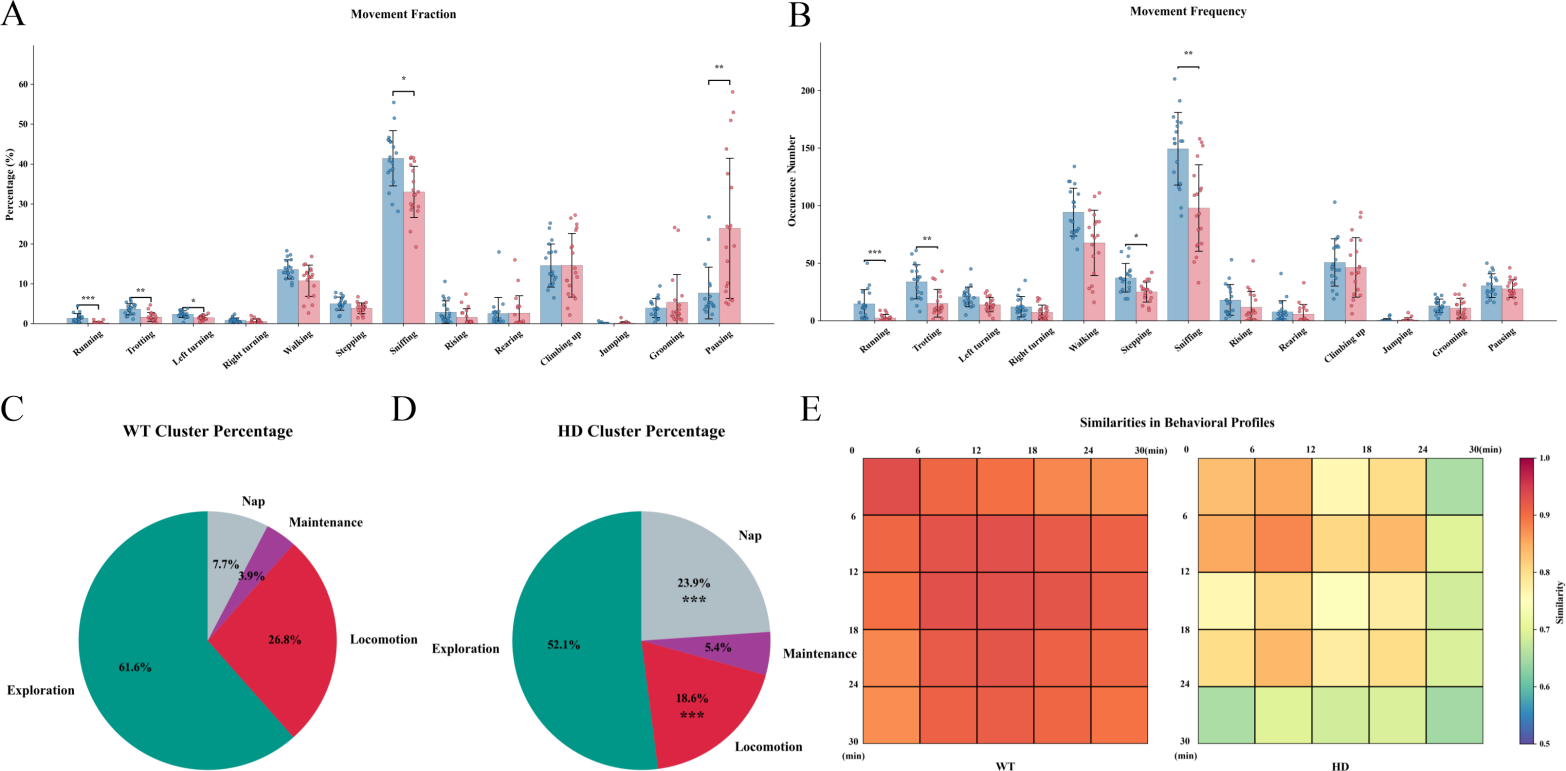
Alterations in movement time observed in HD model mice. **(A)** Movement fraction index in mice, showing values recorded between 20 and 30 minutes. **(B)** Movement frequency index in mice, showing values recorded between 20 and 30 minutes. **(C)** Cluster fraction index in WT mice, showing values recorded between 20 and 30 minutes. **(D)** Cluster fraction index in HD mice, showing values recorded between 20 and 30 minutes. **(E)** Comparison of behavioral similarity between the two groups across different time periods (minutes). (n=19, *<0.05, **<0.01, ***<0.001.) All data were initially subjected to a normality test (Shapiro-Wilk test) and a homogeneity of variance test. If the data satisfied the conditions of normality and homogeneity of variance, one-way analysis of variance and the Bonferroni multiple comparison test were employed for multi - group comparison. For data that were not normally distributed, the non-parametric Mann-Whitney test and Bonferroni-Dunn multiple comparison correction were utilized for statistical analysis.

To thoroughly investigate the dynamic characteristics of motor behaviors in HD mice, we conducted continuous 30-minute observations with behavioral analyses performed in three consecutive 10-minute intervals. During the 0–10 min interval, HD mice exhibited significantly reduced proportions of Running, Trotting, Left turning, and Walking compared to WT mice. In the 10–20 min interval, the proportions of Running, Trotting, and Walking decreased further, while the proportion of Pausing increased. During the 20–30 min interval, the proportions of Running, Trotting, Left turning, and Sniffing were reduced, and Pausing became even more prevalent (**Figure 5A**; **Supplementary Figures 3A, B**). Regarding movement frequency, HD mice showed decreased frequencies of Running, Trotting, Left turning, and Walking during the 0–10 min interval. In the 10–20 min interval, the frequencies of Running, Trotting, Walking, Left turning, and Sniffing were all reduced. By the 20–30 min interval, reductions were observed in the frequencies of Running, Trotting, Stepping, and Sniffing (**Figure 5B**; **Supplementary Figures 3A, B**). At the behavioral pattern level, Locomotion in HD mice progressively declined during the first two intervals (0–10 min and 10–20 min), while Nap behavior gradually emerged and increased in these periods. By the final interval (20–30 min), locomotion remained at a low level, and Nap behavior was further intensified (**Figures 5C, D**; **Supplementary Figures 3C, D**). By comparing the behavioral similarities between two groups of mice across different time periods, it was observed that WT mice maintained a stable and consistent group behavior pattern throughout the entire observation period. In contrast, HD mice exhibited high behavioral consistency only during the initial 0-12-minute interval. Beyond 12 minutes, their behavioral similarity progressively declined, leading to significant divergence (**Figure 5E**). These findings indicate that, even at an early stage, HD mice differ markedly from normal mice in sustained locomotion, exploratory activity, and group coherence. This suggests the presence of early functional impairments in motor capacity and neuropsychiatric function, which may not be detectable using conventional behavioral testing methods.

### 3.5 The alterations of sports area in HD mice models

**Figure 6:**
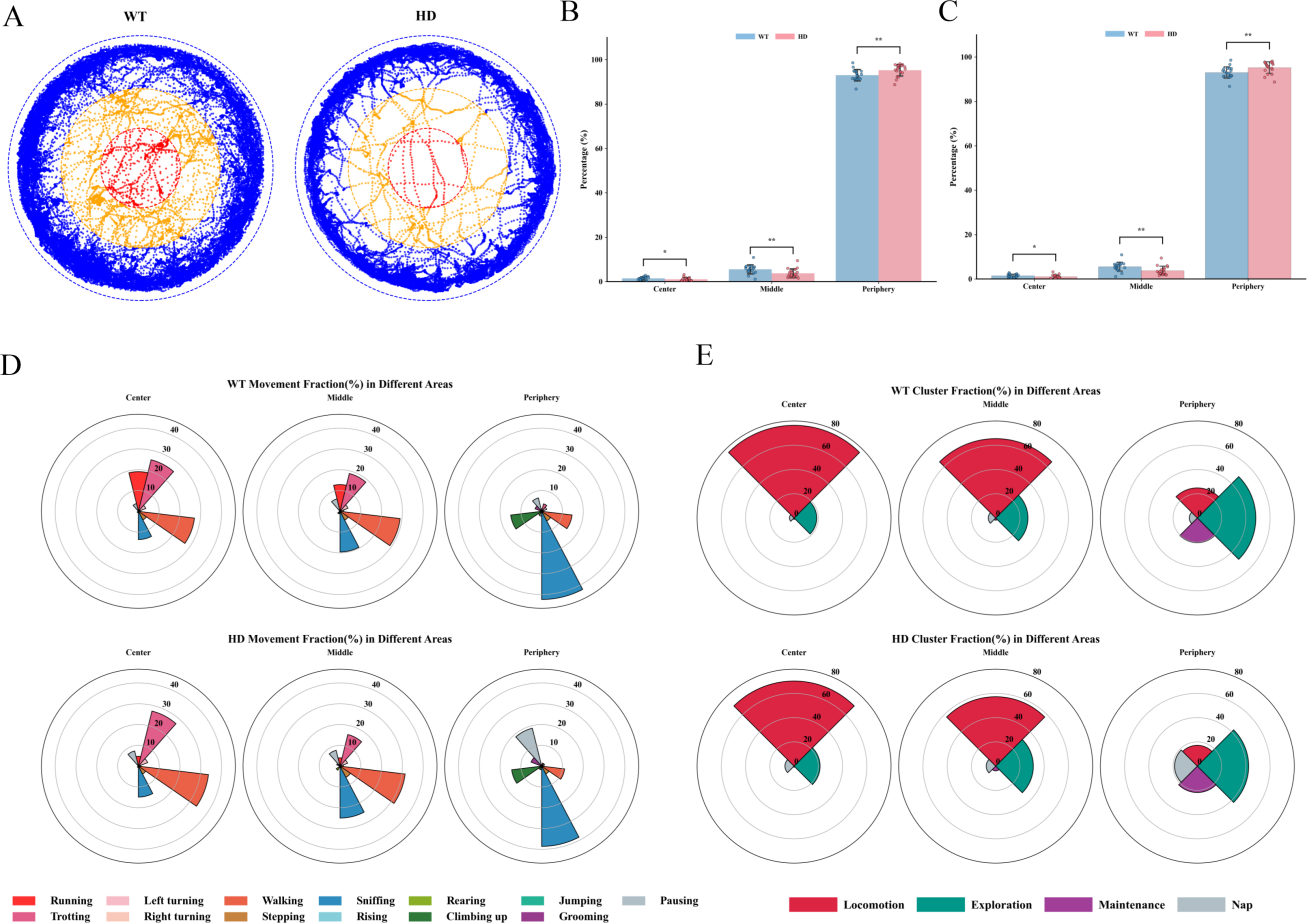
Changes in locomotor activity patterns of HD mice. **(A)** Representative movement trajectories of mice. **(B)** Statistical analysis of the time distribution of mice’s movement across different zones. **(C)** Spatial distribution of movement fraction areas within a 30-minute period. **(D)** Spatial distribution of movement fraction(%) in different areas within a 30-minute period. E Spatial distribution of cluster fraction(%) in different areas within a 30-minute period. (n=19, *<0.05, **<0.01, ***<0.001.) All data were initially subjected to a normality test (Shapiro-Wilk test) and a homogeneity of variance test. If the data satisfied the conditions of normality and homogeneity of variance, one-way analysis of variance and the Bonferroni multiple comparison test were employed for multi - group comparison. For data that were not normally distributed, the non-parametric Mann-Whitney test and Bonferroni-Dunn multiple comparison correction were utilized for statistical analysis.

To thoroughly investigate behavioral and cognitive impairments in early-stage HD mice, we performed a detailed analysis of their sports area. As illustrated in **Supplementary Figure 4A**, the open-field arena was subdivided into three concentric zones: the center, middle, and peripheral regions. During the 30-minute observation period, HD mice exhibited significantly reduced dwell times in the center and middle zones compared to WT controls, while spending significantly more time in the peripheral zone (**Figures 6A–C**). Regarding movement patterns, the proportion of Running in HD mice was decreased in both the center and middle zones. In the periphery, the proportions of active locomotor behaviors-including Running, Trotting, Walking, and Left-turning, were all reduced, whereas the proportion of Pausing was markedly increased (**Figure 6D**). In terms of behavioral category distribution, HD mice showed decreased Locomotion in the middle zone and reduced levels of both Locomotion and Exploration in the peripheral zone, accompanied by a significant increase in Nap (**Figure 6E**). Furthermore, the total travel distance of HD mice was significantly lower than that of the WT group (**Supplementary Figure 4B**). These findings collectively indicate that early-stage HD mice display pronounced abnormalities in motor patterns, motor performance, and exploration-related behaviors.

### 3.6 Motor pattern transitions in HD mice

**Figure 7:**
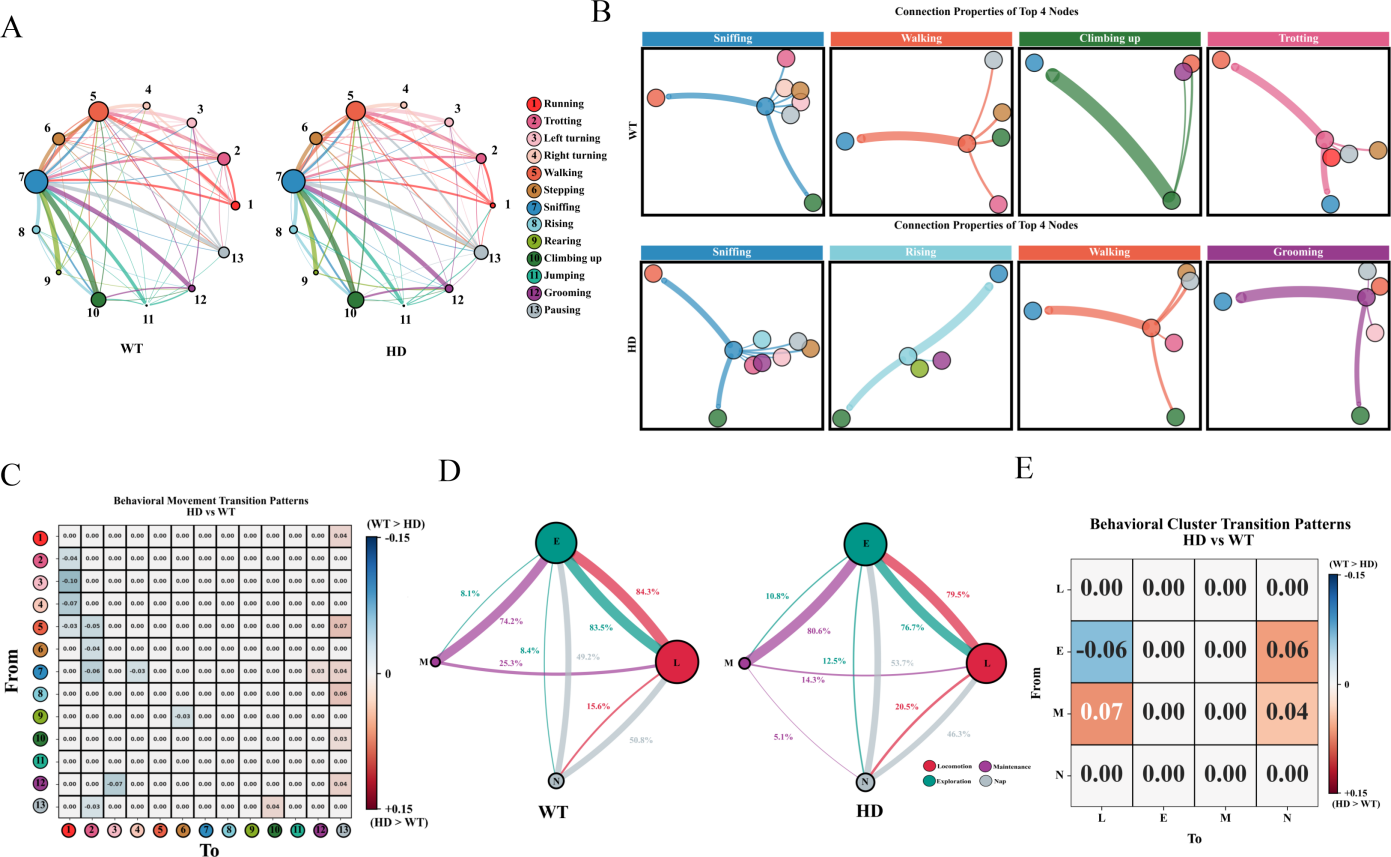
Characteristics of movement transition patterns in HD mice. **(A)** Probability network of movement transitions in mice. **(B)** Central node types and connectivity patterns of movement transitions in mice. **(C)** Differential patterns of 13 types of movement transitions in mice. Red indicates a higher metastasis probability in the HD group relative to the WT group (positive value), whereas blue signifies a lower metastasis probability in the HD group compared to the WT group (negative value). **(D)** Cluster transition patterns in mice. **(E)** Differential analysis of cluster transitions in mice. (n=19, *<0.05, **<0.01, ***<0.001.).

To further investigate the behavioral differences in WT and HD mice, we analyzed the transition patterns among their movements. Each movement segment was represented as a state node, with the node size reflecting the frequency of that behavior. Colored arrows indicated transitions between movements, and arrow thickness corresponded to transition probability. As shown in **Figure 7A**, although the overall transition probabilities among the 13 basic movements were broadly similar between HD and WT mice, HD mice exhibited a higher transition probability into the “Pausing” state. Notably, there were distinct differences in the central nodes and connectivity patterns of movement transitions between the two groups (**Figure 7B**). Analysis of differential transition pathways revealed that WT mice showed a greater propensity to transition into “Running” or “Trotting” across multiple paths, suggesting a stronger tendency toward active locomotor behaviors. In contrast, HD mice displayed significantly increased transition probabilities toward “Pausing,” indicating potential impairments in initiating or sustaining movement (**Figure 7C**). Further examination of movement clusters demonstrated that in HD mice, the transition probabilities from “Exploration” and “Maintenance” transitions to “Nap” were elevated, “Maintenance” transitions to “Locomotion” were increased, while “Exploration” transitions to “Locomotion” were reduced.(**Figures 7D, E**) Additionally, the movement transition entropy in HD mice was significantly lower than in controls (**Supplementary Figure 4C**), implying the behavioral transition patterns in HD mice are relatively simple, with their movements exhibiting pronounced stereotypy, resulting in higher predictability. Collectively, these findings support the conclusion that early-stage HD mice already exhibit motor deficits and aberrant behavioral dynamics that would be undetectable under conventional behavioral assessments.

## 4 Discussion

The present study reveals that precision behavioral analytics uncover early-stage phenotypic anomalies in R6/1 mice at 8 weeks of age, a timepoint preceding overt motor dysfunction detectable with conventional behavioral assessments. These findings challenge the sensitivity of conventional behavioral assays and underscore the necessity of high-resolution phenotyping for detecting premanifest HD pathology. Our approach integrates unsupervised machine learning with high-resolution motion capture to dissect complex behaviors into quantifiable modules (e.g.,Locomotion, Sniffing, Pausing). Key innovations include: Clustering identified 13 distinct behavioral phenotypes, revealing subtle phenotypes (e.g., Sniffing dysregulation) that would be undetectable by manual scoring; Combining kinetic parameters (stride length, gait frequency), spatial distribution (zone occupancy), and transition entropy provided a comprehensive profile of early-stage dysfunction; The LDA classifier achieved an AUC of 0.917, surpassing the predictive value of single-behavior assays. This study establishes precision behavioral phenotyping as a sensitive tool for detecting premanifest HD pathology. The identified signature-reduced locomotion, increased pausing, and spatial avoidance-holds promise as an early diagnostic strategy and the potential utility for preclinically evaluating disease-modifying therapies.

Conventional tests including rotarod, balance beam, and elevated plus maze all failed to discriminate R6/1 from WT mice at 8 weeks, consistent with prior reports demonstrating insensitivity of coarse motor metrics during early HD pathogenesis (8). In the prodromal stage of HD development, patients typically maintain normal daily functioning, exhibiting only subtle motor impairments and a gradual decline in motor performance scores. This observation aligns with the well-documented challenge of detecting early-stage HD in mouse models using conventional behavioral assays, which often lack the sensitivity to capture mild phenotypic changes. These limitations arise from reliance on aggregate performance metrics that obscure nuanced behavioral dynamics. In contrast, clustering of fine-grained movements revealed distinct behavioral fingerprints. For example, HD mice exhibited increased pausing frequency and reduced locomotion fraction in early-stage patients. This discrepancy highlights how automated, multi-dimensional behavioral parsing can resolve subtle phenotypes obscured by manual scoring or low-resolution assays.

HD mice displayed reduced velocity and increased stride length in locomotor movements (Running, Trotting and Walking), paralleling observations of bradykinesia and gait abnormalities in premanifest individuals (18, 19). Reduced upper-body flexion during exploratory behaviors (Sniffing, Rearing) suggested impaired coordination of axial musculature, a hallmark of early HD neuropathology (20, 21). These deficits align with the alteration of frontostriatal connectivity in HD (22).

HD mice displayed increased exploration of the periphery and decreased locomotion in the central zone, a pattern consistent with anxiety-like behavior (23–25). Temporal partitioning revealed an increase in pausing frequency after 10 minutes of testing, implicating early-stage motor fatigue-a precursor to later-stage akinesia (26–28). Notably, movement transition entropy analysis demonstrated stereotyped behavioral repertoires, reflecting compromised executive function and cognitive flexibility. These results agree with previous reports of impaired decision-making and reduced behavioral variability in early HD (29–31).Reductions in sniffing frequency and increased peripheral zone occupancy highlight divergent motivational drives. Sniffing attenuation, defined as a diminished interest in the external environment, aligns with depressed phenotypes in premanifest patients, while spatial avoidance may reflect heightened anxiety or reduced exploratory drive. Notably, the combination of reduced sniffing and peripheral bias parallels observations of environmental detachment in early HD (32–34). HD mice exhibited preferential transitions to pausing from exploratory and maintenance states, suggesting initiation deficits. Reduced exploration-to-locomotion transitions and lower entropy values further indicate behavioral stereotypy. These patterns mirror the executive dysfunction and reduced behavioral flexibility characteristic of HD cognitive decline (35, 36).

While R6/1 mice recapitulate key aspects of early HD, their accelerated pathology contrasts with the slower progression of human disease. Comparative studies with full-length knock-in models (e.g., HdhQ150) are needed to assess generalizability. For example, HD140 mice show hypokinesia at 4 months of age and gait abnormalities only at 12 months of age (37); HdhQ150/Q150 mice show a decline in rotarod performance only at 18 months of age (38); zQ175 homozygous knock-in mice show defects in rotarod and climbing activities at 30 weeks and cognitive deficits at about 1 year of age. Compared with the above-mentioned models, R6 mice have an earlier onset time, obvious motor symptoms, and more sensitive detection methods, and are suitable for rapid drug screening (39).Integration of molecular readouts (e.g., striatal neurofilament light chain) with behavioral data could elucidate structure-function relationships. Future work should extend longitudinal analysis to earlier (e.g., 4–6 weeks) and later stages (e.g., 12–16 weeks) to map the entire progression of behavioral phenotypes.

Several methodological considerations should also be acknowledged when interpreting the present findings. Although the LDA classifier demonstrated strong discriminative performance, the sample size in this study was relatively modest (n = 19 per group), and no independent external validation cohort was included. Leave-one-out cross-validation (LOOCV) was used to maximize data utilization and reduce evaluation bias under small-sample conditions; however, when model development and performance assessment are conducted within the same dataset, the risk of overfitting cannot be fully excluded. Therefore, the classification results should be interpreted as exploratory, primarily indicating that fine-grained behavioral features contain sufficient information to differentiate premanifest HD mice from controls, rather than representing a fully generalizable predictive model. Future studies incorporating larger sample sizes, multi-center replication, and independent validation datasets will be essential to further evaluate model robustness and translational potential.

In addition, this study involved a large number of behavioral metrics, time-window analyses, and spatial distribution measurements, which increases the potential risk of false-positive findings due to multiple comparisons. To address this issue, Bonferroni-Dunn correction was consistently applied throughout the statistical analyses. It is important to note that Bonferroni-type corrections are generally considered conservative approaches: while they effectively reduce the probability of Type I errors (false positives), they may also increase the risk of Type II errors (false negatives). Thus, our statistical framework favored stricter significance criteria rather than inflating positive findings. Moreover, the analyses followed a hierarchical, hypothesis-guided structure, progressing from broader behavioral clusters to functionally meaningful subtypes, with convergent support drawn from kinematic, temporal, spatial, and transition-level features. Nevertheless, future studies may benefit from incorporating additional dimensionality-reduction and multivariate integration strategies to construct low-dimensional composite behavioral phenotypes. In addition to traditional principal component analysis (PCA), nonlinear manifold learning approaches such as Uniform Manifold Approximation and Projection (UMAP) may offer advantages in preserving both local and global structures of high-dimensional behavioral features. Such approaches could help reduce the statistical burden associated with multiple comparisons while enhancing the robustness and interpretability of behavioral phenotyping.

Precision behavioral analytics reveal that R6/1 mice exhibit early-stage behavioral anomalies spanning motor, psychiatric, and cognitive domains. By deconstructing complex behaviors into quantifiable modules, we identified subtle phenotypes-sniffing deficits, spatial avoidance, and behavioral stereotypy-that align with clinical HD manifestations. This framework not only advances our understanding of prodromal HD biology, but also provides a scientific foundation for the future development of precision behavioral analysis systems that may serve as sensitive tools for early clinical detection and for evaluating therapeutic strategies targeting presymptomatic neuronal abnormalities.

## Data Availability

The raw data supporting the conclusions of this article will be made available by the authors, without undue reservation.

## Supplementary material

Supplementary Figure 1 A schematic diagram of this study. **(A)** Schematic diagram of the research device. **(B)** Schematic diagram of the research timeline.

Supplementary Figure 2 Refinement of the movement atlas in HD mice.

Supplementary Figure 3 Movement time Alterations in HD model mice. **(A)** Movement fraction and frequency index in mice, showing values recorded between 0 and 10 minutes. **(B)** Movement fraction and frequency index in mice, showing values recorded between 10 and 20 minutes. **(C)** Cluster fraction index in mice, showing values recorded between 0 and 10 minutes. D Cluster fraction index in mice, showing values recorded between 10 and 20 minutes. (n=19, *<0.05, **<0.01, ***<0.001.).

Supplementary Figure 4 HD mice exhibit altered movement trajectories and movement transitions. **(A)** Schematic diagram of the movement area in mice. **(B)** Comparative analysis of total movement distances between HD and WT mice. **(C)** Movement transition entropy values in HD and WT mice. (n=19, *<0.05, **<0.01, ***<0.001.).

## References

[1] Jimenez-Sanchez M Licitra F Underwood BR Rubinsztein DC . Huntington’s disease: mechanisms of pathogenesis and therapeutic strategies. Cold Spring Harb Perspect Med. (2017) 7:1–27. doi: 10.1101/cshperspect.a024240, PMID: 27940602 PMC5495055

[2] Tabrizi SJ Schobel S Gantman EC Mansbach A Borowsky B Konstantinova P . A biological classification of Huntington’s disease: the Integrated Staging System. Lancet Neurol. (2022) 21:632–44. doi: 10.1016/S1474-4422(22)00120-X, PMID: 35716693

[3] Ross CA Tabrizi SJ . Huntington’s disease: from molecular pathogenesis to clinical treatment. Lancet Neurol. (2011) 10:83–98. doi: 10.1016/S1474-4422(10)70245-3, PMID: 21163446

[4] Etxeberria-Rekalde E Alzola-Aldamizetxebarria S Flunkert S Hable I Daurer M Neddens J . Quantification of huntington’s disease related markers in the R6/2 mouse model. Front Mol Neurosci. (2020) 13:617229. doi: 10.3389/fnmol.2020.617229, PMID: 33505246 PMC7831778

[5] Yamin HG Menkes-Caspi N Stern EA Cohen D . Age-dependent degradation of locomotion encoding in huntington’s disease R6/2 model mice. J Huntingtons Dis. (2021) 10:391–404. doi: 10.3233/JHD-210492, PMID: 34420979 PMC8609681

[6] Ehrnhoefer DE Butland SL Pouladi MA Hayden MR . Mouse models of Huntington disease: variations on a theme. Dis Models Mech. (2009) 2:123–9. doi: 10.1242/dmm.002451, PMID: 19259385 PMC2650190

[7] Brooks SP Jones L Dunnett SB . Comparative analysis of pathology and behavioral phenotypes in mouse models of Huntington’s disease. Brain Res Bull. (2012) 88:81–93. doi: 10.1016/j.brainresbull.2011.10.002, PMID: 22004616

[8] Ouwerkerk J Feleus S van der Zwaan KF Li Y Roos M van Roon-Mom WMC . Machine learning in Huntington’s disease: exploring the Enroll-HD dataset for prognosis and driving capability prediction. Orphanet J Rare Dis. (2023) 18:218. doi: 10.1186/s13023-023-02785-4, PMID: 37501188 PMC10375780

[9] Ye J Xu Y Huang K Wang X Wang L Wang F . Hierarchical behavioral analysis framework as a platform for standardized quantitative identification of behaviors. Cell Rep. (2025) 44:115239. doi: 10.1016/j.celrep.2025.115239, PMID: 40010299

[10] Huang K Han Y Chen K Pan H Zhao G Yi W . A hierarchical 3D-motion learning framework for animal spontaneous behavior mapping. Nat Commun. (2021) 12:2784. doi: 10.1038/s41467-021-22970-y, PMID: 33986265 PMC8119960

[11] McGuirk TE Perry ES Sihanath WB Riazati S Patten C . Feasibility of markerless motion capture for three-dimensional gait assessment in community settings. Front Hum Neurosci. (2022) 16:867485. doi: 10.3389/fnhum.2022.867485, PMID: 35754772 PMC9224754

[12] Li JY Popovic N Brundin P . The use of the R6 transgenic mouse models of Huntington’s disease in attempts to develop novel therapeutic strategies. NeuroRx. (2005) 2:447–64. doi: 10.1602/neurorx.2.3.447, PMID: 16389308 PMC1144488

[13] Han Y Huang K Chen K Pan H Ju F Long Y . MouseVenue3D: A markerless three-dimension behavioral tracking system for matching two-photon brain imaging in free-moving mice. Neurosci Bull. (2022) 38:303–17. doi: 10.1007/s12264-021-00778-6, PMID: 34637091 PMC8975979

[14] Han Y Chen K Wang Y Liu W Wang Z Wang X . Multi-animal 3D social pose estimation, identification and behaviour embedding with a few-shot learning framework. Nat Mach Intell. (2024) 6:48–61. doi: 10.1038/s42256-023-00776-5, PMID: 41775740

[15] Jingjing L Ye J Ji C Ren W He Y Xu F . Mapping the behavioral signatures of shank3b mice in both sexes. Neurosci Bull. (2024) 40:1299–314. doi: 10.1007/s12264-024-01237-8, PMID: 38900384 PMC11365888

[16] Podlacha M Pierzynowska K Gaffke L Jerzemowska G Piotrowska E Węgrzyn G . Behavioral- and blood-based biomarkers for Huntington’s disease: Studies on the R6/1 mouse model with prospects for early diagnosis and monitoring of the disease. Brain Behav Immun Health. (2022) 23:100482. doi: 10.1016/j.bbih.2022.100482, PMID: 35799674 PMC9253406

[17] Menalled LB Chesselet MF . Mouse models of Huntington’s disease. Trends Pharmacol Sci. (2002) 23:32–9. doi: 10.1016/S0165-6147(00)01884-8, PMID: 11804649

[18] Wiprich MT Altenhofen S Gusso D Vasques RDR Zanandrea R Kist LW . Modulation of adenosine signaling reverses 3-nitropropionic acid-induced bradykinesia and memory impairment in adult zebrafish. Prog Neuropsychopharmacol Biol Psychiatry. (2022) 119:110602. doi: 10.1016/j.pnpbp.2022.110602, PMID: 35843370

[19] Anderson DG Krause A Margolis RL . Huntington Disease-Like 2. (2004) In: Adam MP Bick S Mirzaa GM Pagon RA Wallace SE Amemiya A , editors. GeneReviews® [Internet]. Seattle (WA): University of Washington, Seattle; 1993–2026.

[20] Rüb U Seidel K Heinsen H Vonsattel JP den Dunnen WF Korf HW . Huntington’s disease (HD): the neuropathology of a multisystem neurodegenerative disorder of the human brain. Brain Pathol. (2016) 26:726–40. doi: 10.1111/bpa.12426, PMID: 27529157 PMC8029421

[21] Rosas HD Salat DH Lee SY Zaleta AK Hevelone N Hersch SM . Complexity and heterogeneity: what drives the ever-changing brain in Huntington’s disease? Ann N Y Acad Sci. (2008) 1147:196–205. doi: 10.1196/annals.1427.034, PMID: 19076442 PMC2813569

[22] Padron-Rivera G Romero-Molina AO Diaz R Vaca-Palomares I Ochoa-Morales A Romero-Rebollar C . Frontostriatal circuits alterations associated with cognitive flexibility deterioration in huntington’s disease. Neurodegener Dis. (2022) 22:24–8. doi: 10.1159/000526778, PMID: 36067733

[23] Noureldeen ME Shahin NN Amin HAA El-Sawalhi MM Ghaiad HR . Parthenolide ameliorates 3-nitropropionic acid-induced Huntington’s disease-like aberrations via modulating NLRP3 inflammasome, reducing microglial activation and inducing astrocyte shifting. Mol Med. (2024) 30:158. doi: 10.1186/s10020-024-00917-5, PMID: 39327568 PMC11425901

[24] Peres DS Viero FT Rodrigues P de Barros Bernardes L da Silva NAR Lima IR . Characterization of depression- and anxiety-like behaviours in a mouse model of relapsing-remitting multiple sclerosis. J Neuroimmune Pharmacol. (2023) 18:235–47. doi: 10.1007/s11481-023-10080-z, PMID: 37526817

[25] Xiong Y Zhu J He Y Qu W Huang Z Ding F . Sleep fragmentation reduces explorative behaviors and impairs motor coordination in male mice. J Neurosci Res. (2024) 102:e25268. doi: 10.1002/jnr.25268, PMID: 38284850

[26] Miranda DR Reed E Jama A Bottomley M Ren H Rich MM . Mechanisms of altered skeletal muscle action potentials in the R6/2 mouse model of Huntington’s disease. Am J Physiol Cell Physiol. (2020) 319:C218–c232. doi: 10.1152/ajpcell.00153.2020, PMID: 32432924 PMC7468886

[27] Wang HB Loh DH Whittaker DS Cutler T Howland D Colwell CS . Time-restricted feeding improves circadian dysfunction as well as motor symptoms in the Q175 mouse model of huntington’s disease. eNeuro. (2018) 5:1–17. doi: 10.1523/ENEURO.0431-17.2017, PMID: 29302618 PMC5752678

[28] Miranda DR Wong M Romer SH McKee C Garza-Vasquez G Medina AC . Progressive Cl- channel defects reveal disrupted skeletal muscle maturation in R6/2 Huntington’s mice. J Gen Physiol. (2017) 149:55–74. doi: 10.1085/jgp.201611603, PMID: 27899419 PMC5217084

[29] El Massioui N Lamirault C Yagüe S Adjeroud N Garces D Maillard A . Impaired decision making and loss of inhibitory-control in a rat model of huntington disease. Front Behav Neurosci. (2016) 10:204. doi: 10.3389/fnbeh.2016.00204, PMID: 27833538 PMC5080295

[30] Scheres A Oosterlaan J Sergeant JA . Response execution and inhibition in children with AD/HD and other disruptive disorders: the role of behavioral activation. J Child Psychol Psychiatry. (2001) 42:347–57. doi: 10.1111/1469-7610.00728, PMID: 11321204

[31] Cao LX Yin JH Du G Yang Q Huang Y . Identifying and verifying Huntington’s disease subtypes: Clinical features, neuroimaging, and cytokine changes. Brain Behav. (2024) 14:e3469. doi: 10.1002/brb3.3469, PMID: 38494708 PMC10945031

[32] Clifford JJ Drago J Natoli AL Wong JY Kinsella A Waddington JL . Essential fatty acids given from conception prevent topographies of motor deficit in a transgenic model of Huntington’s disease. Neuroscience. (2002) 109:81–8. doi: 10.1016/S0306-4522(01)00409-2, PMID: 11784701

[33] Stoker TB Mason SL Greenland JC Holden ST Santini H Barker RA . Huntington’s disease: diagnosis and management. Pract Neurol. (2022) 22:32–41. doi: 10.1136/practneurol-2021-003074, PMID: 34413240

[34] Ghosh R Tabrizi SJ . Clinical features of huntington’s disease. Adv Exp Med Biol. (2018) 1049:1–28. doi: 10.1007/978-3-319-71779-1_1, PMID: 29427096

[35] Walker FO . Huntington’s disease. Lancet. (2007) 369:218–28. doi: 10.1016/S0140-6736(07)60111-1, PMID: 17240289

[36] Wilton DK Mastro K Heller MD Gergits FW Willing CR Fahey JB . Microglia and complement mediate early corticostriatal synapse loss and cognitive dysfunction in Huntington’s disease. Nat Med. (2023) 29:2866–84. doi: 10.1038/s41591-023-02566-3, PMID: 37814059 PMC10667107

[37] Menalled LB Sison JD Dragatsis I Zeitlin S Chesselet MF . Time course of early motor and neuropathological anomalies in a knock-in mouse model of Huntington’s disease with 140 CAG repeats. J Comp Neurol. (2003) 465:11–26. doi: 10.1002/cne.10776, PMID: 12926013

[38] Woodman B Butler R Landles C Lupton MK Tse J Hockly E . The Hdh(Q150/Q150) knock-in mouse model of HD and the R6/2 exon 1 model develop comparable and widespread molecular phenotypes. Brain Res Bull. (2007) 72:83–97. doi: 10.1016/j.brainresbull.2006.11.004, PMID: 17352931

[39] McLean FH Monteiro O Lelos MJ Ekkunagul T Spicer RM Rybnicek J . Development of cognitive, motor, metabolic, and mutant huntingtin aggregation in the zQ175 mouse model of Huntington’s disease. Sci Rep. (2025) 15:34563. doi: 10.1038/s41598-025-17956-5, PMID: 41044411 PMC12494737

