# Supplementary figures and images for: 3D-AI mouse behavior analysis system has the capability to detect abnormalities in R6/1 model mice with Huntington’s disease during the pre-symptomatic phase
Source: Front Psychiatry. 2026 Mar 10;17:1749543. doi: 10.3389/fpsyt.2026.1749543 (PMC13008838; doi:10.3389/fpsyt.2026.1749543)

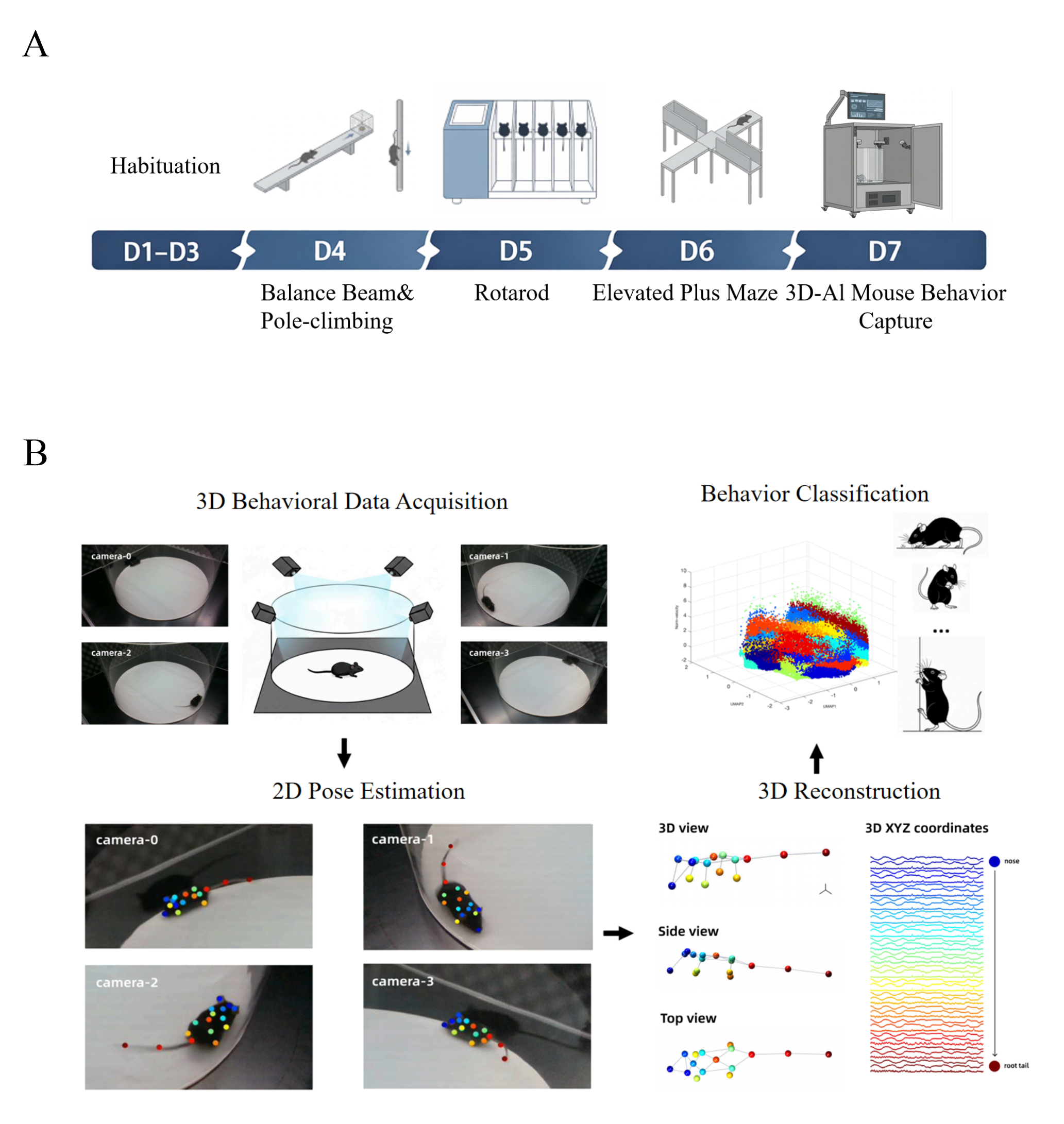

Supplement: Supplementary Figure 1 — A schematic diagram of this study. (A) Schematic diagram of the research device. (B) Schematic diagram of the research timeline. [file Image1.tif]

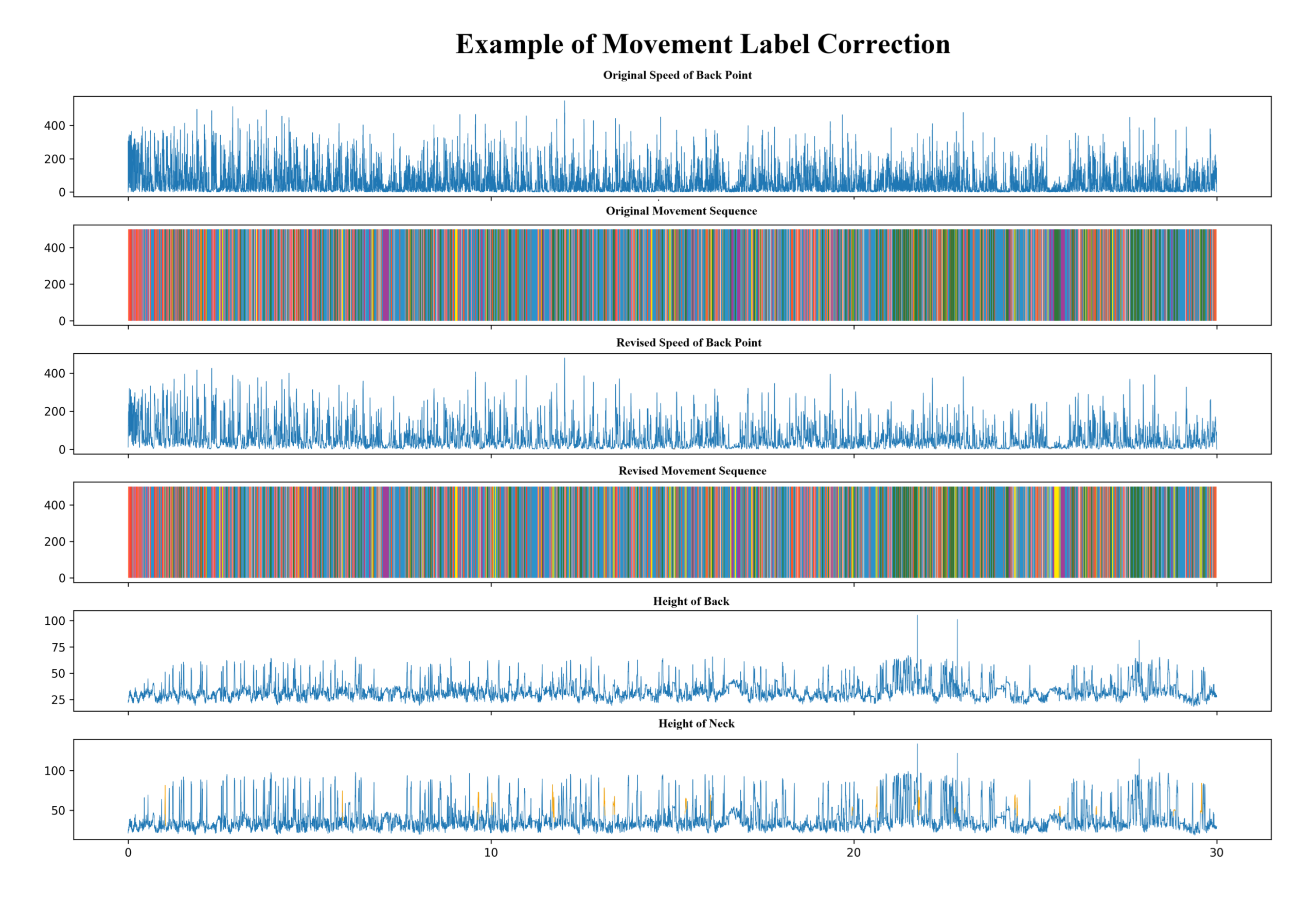

Supplement: Supplementary Figure 2 — Refinement of the movement atlas in HD mice. [file Image2.tif]

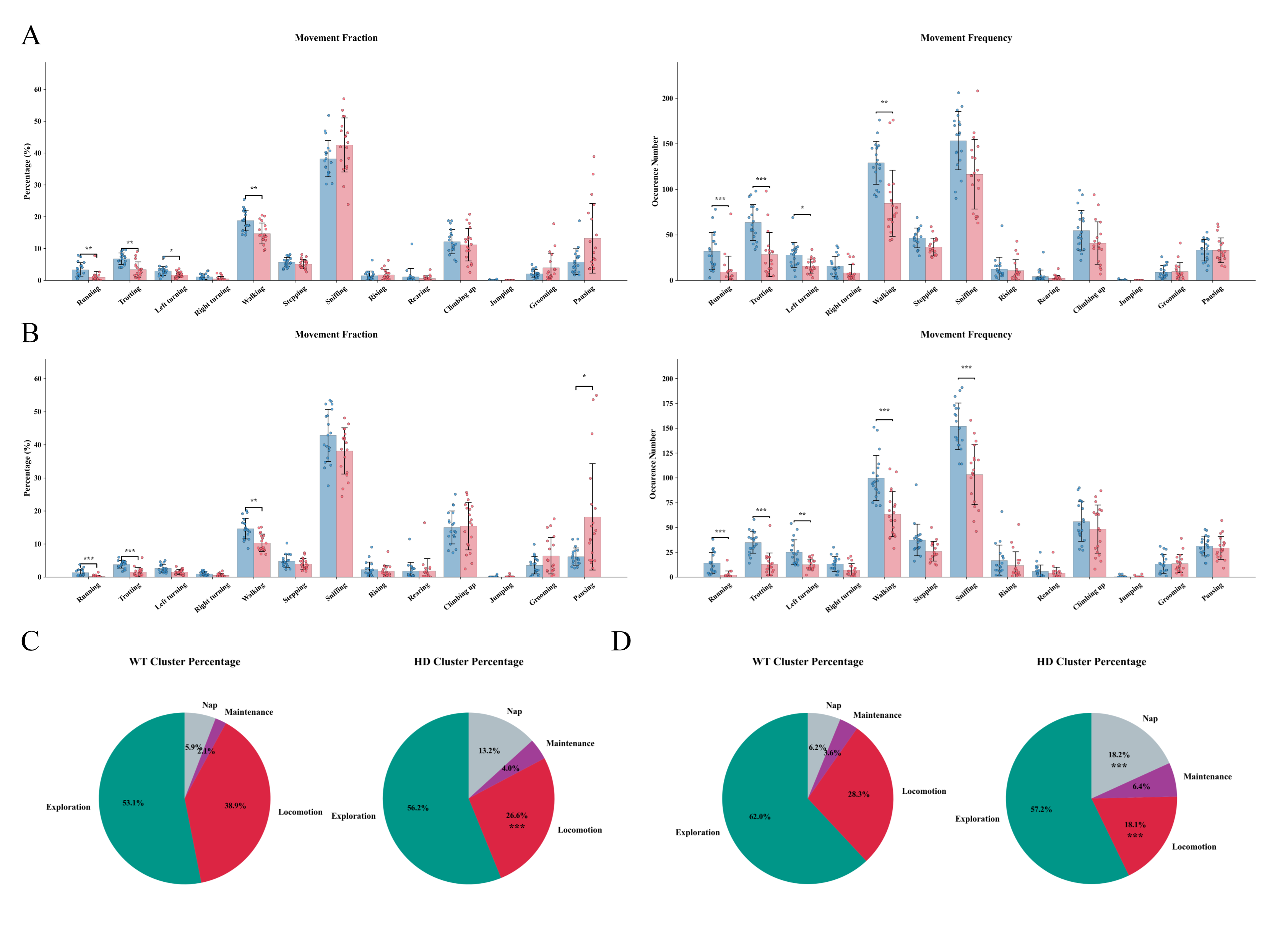

Supplement: Supplementary Figure 3 — Movement time Alterations in HD model mice. (A) Movement fraction and frequency index in mice, showing values recorded between 0 and 10 minutes. (B) Movement fraction and frequency index in mice, showing values recorded between 10 and 20 minutes. (C) Cluster fraction index in mice, showing values recorded between 0 and 10 minutes. D Cluster fraction index in mice, showing values recorded between 10 and 20 minutes. (n=19, *<0.05, **<0.01, ***<0.001.). [file Image3.tif]

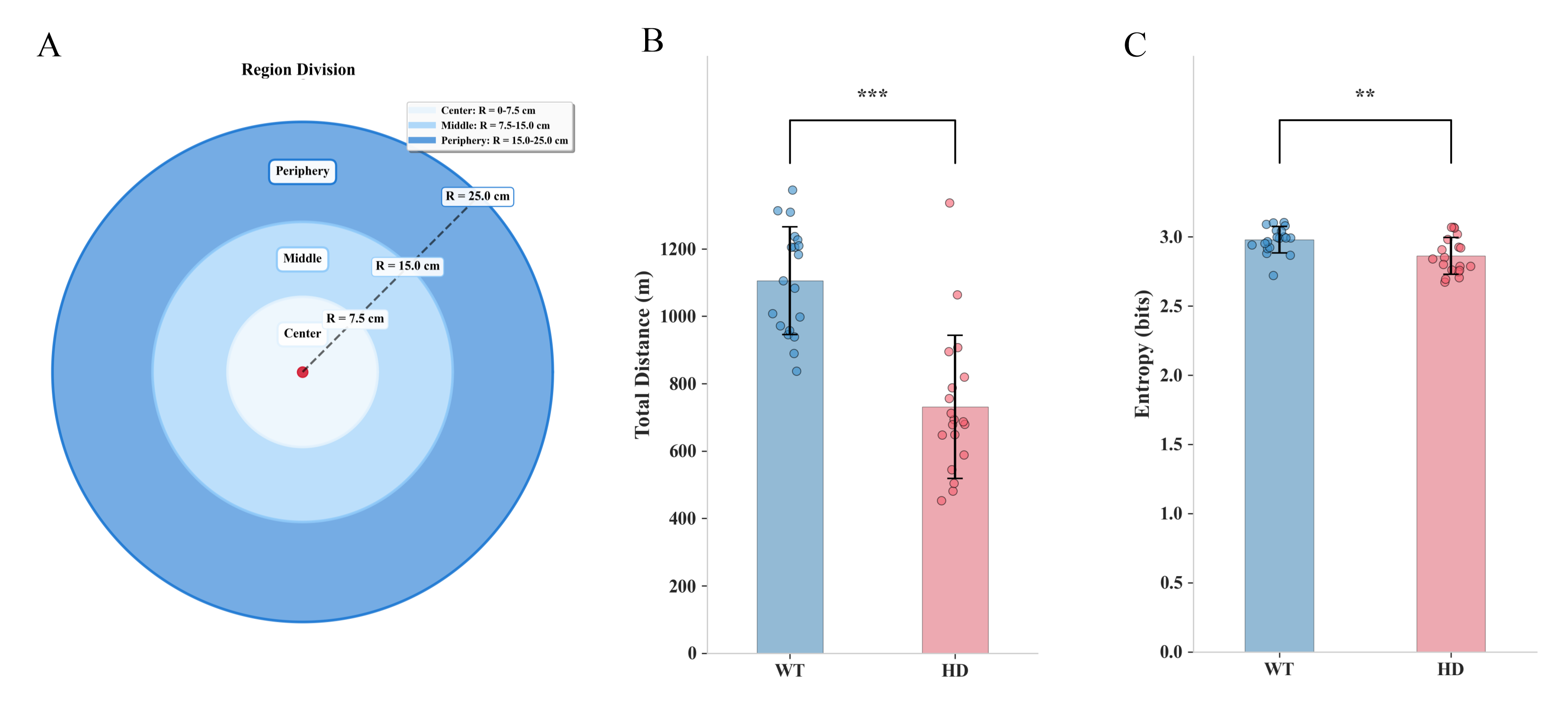

Supplement: Supplementary Figure 4 — HD mice exhibit altered movement trajectories and movement transitions. (A) Schematic diagram of the movement area in mice. (B) Comparative analysis of total movement distances between HD and WT mice. (C) Movement transition entropy values in HD and WT mice. (n=19, *<0.05, **<0.01, ***<0.001.). [file Image4.tif]
